# Extreme value analysis of wind droughts in Great Britain

**DOI:** 10.1016/j.renene.2023.119847

**Published:** 2024-02

**Authors:** Panit Potisomporn, Thomas A.A. Adcock, Christopher R. Vogel

**Affiliations:** Department of Engineering Science, University of Oxford, Parks Road, Oxford, OX1 3PJ, United Kingdom

**Keywords:** Wind energy, Wind droughts, Extreme value analysis, Pooling procedures

## Abstract

Due to the growing proportion of wind energy in Great Britain’s energy mix, prolonged periods of low wind power generation have become a significant challenge for decarbonising the electricity system. As such, characterising drought severity and duration is important for ensuring the reliability of the electricity system. Employing concepts derived from hydrology, an extreme value analysis was carried out on wind drought events in Great Britain based on 72 years of ERA5 reanalysis data. The application of pooling procedures was found to be beneficial in robustly identifying wind droughts in cases where the capacity factor is not constantly below an arbitrary threshold. The sequent peak algorithm pooling was found to have particular relevance for electricity systems where energy storage technologies are used to compensate for low wind power generation. The Pearson-III distribution was identified as a suitable model to represent extreme wind droughts, while the Lognormal and Generalised Pareto distributions are also viable alternatives. Sustained periods of low wind power generation with a duration of 14 days were estimated to have a return period of five years and the longest event on record of approximately 26 days is expected to occur once every 100 years. The investigation of these wind droughts from a hydrological perspective has thus shown that they may not be particularly rare occurrences.

## Highlights

• Pooling methods can characterise wind droughts as periods of overall low energy generation.
• Sequent Peak Algorithm is able to identify the longest wind droughts.
• Pearson-III distribution is a suitable model for representing extreme wind droughts.
• Wind droughts lasting two weeks are estimated to have a return period of five years.

## 1 Introduction

In March 2021, Great Britain (GB) experienced one of the longest cold calm spells in over a decade; the national wind farm fleet operated at 11% of its rated capacity for eleven consecutive days during a period where low temperatures drove up heating and electricity demand [1]. In response, dispatchable fossil-fuelled generation was increased to compensate for the reduction in wind power generation. Although such wind droughts, which stem from extensive high-pressure systems [2], are uncommon, they are regarded as one of the biggest challenges in fully decarbonising the GB electricity system. Therefore, as the proportion of wind energy in the GB energy mix increases, so does the need to characterise the risk of wind droughts and their implications for the future and resilience of the GB electricity system.

**Fig. 1:**
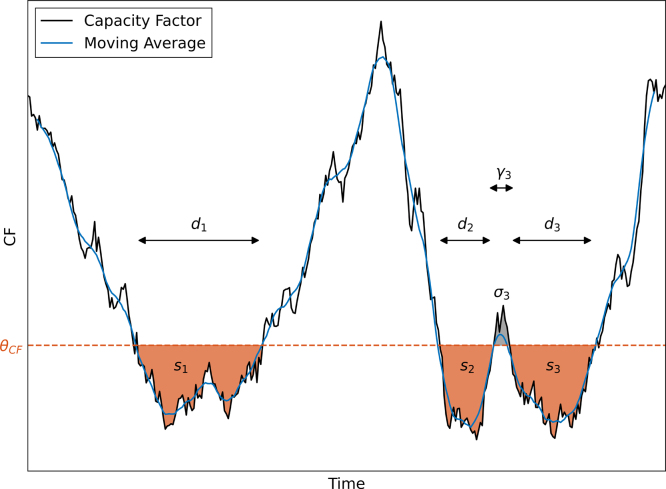
Illustrations of wind generation drought events (coloured in orange), as defined by the threshold level and their drought characteristics: duration $d_{i}$, severity $s_{i}$, excess time $\gamma_{i}$, and excess severity $\sigma_{i}$.

Defining the wind drought period is an important first step in characterising drought frequency and duration. Fig. 1 illustrates a time series of capacity factor CF, with droughts indicated in colour as periods below the CF threshold, $\theta_{CF}$. One approach is to define a drought as a period where the instantaneous power output is continuously below a specified threshold [3], [4]. Alternatively, wind droughts can be defined in terms of being below a specified threshold of the moving average of the wind energy time series, which can result in identification of longer drought periods [5]. However, this approach is highly reliant on the choice of the averaging interval and may also introduce dependency between drought events.

As illustrated in Fig. 1, both approaches can be sensitive to the choice of wind speed or power threshold, in particular for small values, which may affect statistical descriptions of drought likelihood and severity. While both methods identify drought 1 as a single event, a short period $\gamma_{3}$ where $CF>\theta_{CF}$ separates droughts 2 and 3. Whether these are usefully characterised as separate droughts or as part of one longer drought depends on the objectives of the analysis. Periods where instantaneous generation is below a threshold may be useful to characterise individually when dispatchable generation is used to compensate for low wind power, whereas grouping nearby events to calculate overall wind power deficits may be more appropriate when describing the need for energy storage technologies.

Hydrology offers valuable insights for drought characterisation. Analogous to wind droughts, hydrological droughts are prolonged periods of low streamflows that may have significant impacts on water supply. The analogy further extends in that streamflow and wind resource time series, from which these droughts are identified, are both zero-limited, non-normally-distributed, and at a particular time scale, non-stationary [6], [7]. By leveraging these conceptual and statistical similarities, several well-researched tools and techniques which have been applied in a multitude of hydrological droughts studies, can be employed in the investigation of wind droughts.

Statistical descriptions of rare drought events have been made using extreme value analysis, a well-developed statistical methodology that focuses on modelling probabilities associated with rare events that display a substantial deviation from the median of a population [8]. Extreme values are extracted from the sample data, modelled with a distribution, and the return period is calculated from the distribution. An advantage of associating the sampled data with an extreme value distribution is that it allows inferences to be drawn extending beyond those observed in the sampled events themselves. This process has been applied to the investigation of hydrological droughts; see e.g., Zelenhasić and Salvai, Tallaksen et al. [9], [10], and Shiau [11]. Hydrological extreme value analyses are often complemented with other hydrology-based techniques. For example, runs analysis [12] is commonly used to identify periods of droughts from a streamflow time series and pooling procedures [10], [13], [14] are regularly employed to address the issues of minor and mutually dependent droughts [15].

Extreme value analyses have been conducted on wind droughts in the North Sea and Ireland by Patlakas et al. [16] and Leahy and McKeogh [17] respectively, who similarly found low wind speed events lasting over four days to have a return period of around ten years. To incorporate electricity demand and the influence of climate change into the analysis, Dawkins and Rushby [18] conducted an extreme value analysis on historical, simulated, and projected electricity demand net of wind generation (demand-net-wind) time series and found that periods of low wind power generation during peak demand with a duration of eleven days in winter are estimated to have a return period of ten years.

This study focuses on how the robustness of wind drought analysis can be improved. Pooling procedures to group related wind drought periods are compared with reference to the derived drought severity and duration statistics. Fitted theoretical distributions are used for extreme value analysis in order to allow extrapolation of results to longer return periods than can be achieved empirically from observations alone. These are used to conduct a case study on the frequency and risk of wind droughts in GB.

## 2 Data sources and processing

In this study, an extreme value analysis of wind droughts was carried out on a GB-aggregated capacity factor time series based on wind speed data from the European Centre for Medium-Range Weather Forecast’s Reanalysis-5 (ERA5) data for 1950–2022, and the 2022 distribution and capacity of wind farms in Great Britain. The steps to derive the aggregated CF series are summarised below, with full details available in Supplementary Information.

### 2.1 Reanalysis data description and evaluation

Released in 2019, ERA5 [19] is the latest generation reanalysis model produced by ECMWF using a four-dimensional variational (4D-VAR) assimilation scheme. With a spatial resolution of 0.25${}^{\circ }$ latitude–longitude (approximately 27.5 km) and a maximum temporal resolution of 1-hourly time steps, ERA5 offers the highest spatiotemporal resolution out of all global reanalyses. Furthermore, ERA5’s extension back to 1950 means it also provides one of the longest temporal coverages relative to other global reanalyses of comparable spatiotemporal resolution. This is of particular importance within the context of this study as the rarity of extreme wind droughts necessitates a long dataset that captures inter-decadal variability in wind speeds. For example, the average wind speed in GB during 1960–1980 was significantly lower than that in the following two decades [20].

Potisomporn et al. [21] evaluated ERA5 10 m wind speed against in-situ surface wind speed measurements from 205 onshore and offshore observation stations around GB from the period 1997–2021. It was found that ERA5 10 m wind speed exhibits biases in mean wind speed of 0.166 m/s and −0.136 m/s for the onshore and offshore domains respectively, while also underestimating wind speed standard deviation by over 0.35 m/s. The study also reported that the frequency of short-duration low wind speed events (defined as periods where the wind speed is below a typical wind turbine cut-in speed, 4 m/s, for less than 12 h) is significantly underestimated in ERA5. This was attributed to ERA5 not adequately capturing hourly wind variability arising from small-scale atmospheric fluctuations, hence its inability to capture small, frequent drops in wind speed that give rise to short duration events. However, longer low wind speed events, which are the focus of the present study, were found to be more accurately represented by ERA5 and corroborated by Cannon et al. [3] for the Modern-Era Retrospective analysis for Research and Applications 2 dataset.

In this study, ERA5 wind speed data were bias corrected through mean–variance scaling using the bias levels reported by Potisomporn et al. [21], taking into account ERA5’s inherent limitations in capturing short-duration low wind speed events.

### 2.2 GB wind farms and spatial interpolation

**Fig. 2:**
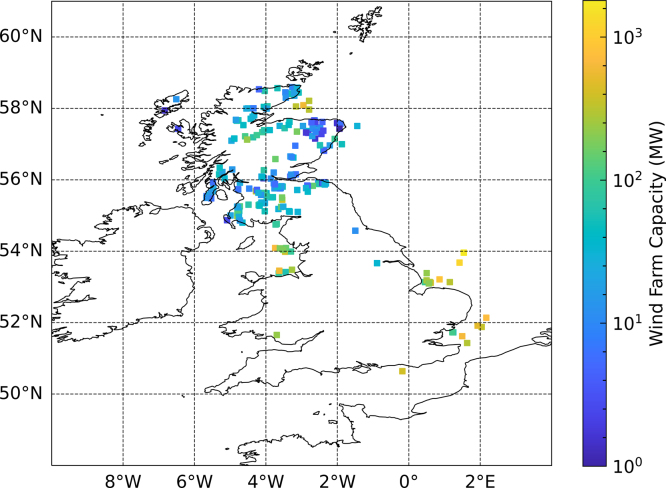
2022 wind farm distribution and installed capacity in Great Britain based on National Grid and Elexon datasets [22], [23], [24].

GB wind farms in this study are based on the 2022 distribution of wind farms, assumed to be constant throughout the study period. The distribution was sourced from National Grid’s Transmission Entry Capacity register [22], National Grid’s Embedded register [23], and Elexon [24]. Together, these datasets comprise 222 onshore and offshore non-embedded wind farms (metred and connected to the transmission network), as well as embedded wind farms (unmetered and directly connected to the distribution network), which represent 86% of installed wind power capacity in GB, and are illustrated in Fig. 2. The gridded ERA5 10 m wind speed was bi-linearly interpolated to the location of each wind farm.

### 2.3 Extrapolation of wind speed to hub height

Due to the widely-recognised limitations of the conventional logarithmic wind profile at low wind speeds [18], [25], in this study a Multi-Layer Perceptron Regression (MLPR) model was used to vertically extrapolate 10 m wind speed data to 100 m at each wind farm. The model was designed to use meteorological variables relevant to Monin–Obukhov’s similarity theory that are available in ERA5 as input features, such as boundary layer height and friction velocity, as well as geographical and temporal features. The MLPR model was trained on 364 regularly sampled ERA5 grid points within the study domain and two randomly selected years, at hourly time steps, representing a training set of over 12 million data points.

### 2.4 Power output modelling

Power curves provided by turbine manufacturers are a simple means to convert wind speed to power output. However, they do not capture the complexities of the relationship between wind speed and farm power output which is influenced by factors at a turbine level (e.g., hysteresis effects, decline in turbine performance with age) and at a farm level (e.g., wake effects, maintenance, spatial variability). Hence, a farm power curve model was developed and employed in this study to take these effects into account. The farm power curve was derived from representative turbine power curves using a five-parameter logistic function, which has been found to show substantial accuracy in estimating power output from a turbine [26]. Farm-level effects, namely wake losses and farm availability, were then estimated from measured wind farm power output data [27]. Finally, wind farm energetic availabilities obtained from Cevasco et al. [28] were applied to the power curve.

### 2.5 GB-aggregated CF and validation

**Fig. 3:**
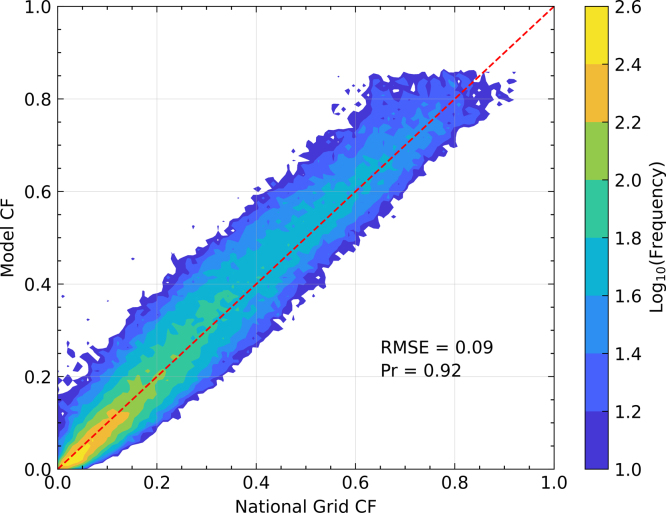
Modelled GB-aggregated capacity factor plotted against recorded capacity factor from National Grid ESO [24].

The GB-aggregated and capacity weighted CF was calculated for all N=222 wind farms through:

(1) $$CF\left(t\right)=\frac{1}{C}\overset{N}{\underset{i}{\sum }}cf_{i}\left(t\right)c_{i},$$

where $C=\sum_{i}c_{i}$ is the total installed capacity of GB wind farms, $cf_{i}$ the capacity factor of a single farm, and $c_{i}$ the farm’s installed capacity. The resultant GB-aggregated CF was then validated against CF calculated from wind power generation data from National Grid [24], and is presented in Fig. 3. While the CF RMSE value of 0.09 is not insignificant, the two time series are highly correlated and visually display reasonable agreement. Moreover, a sensitivity analysis conducted with respect to the distribution of wind farms (not presented) showed that the CF time series is very weakly sensitive to modest changes to the wind farm distribution, thereby providing greater confidence in using the resultant time series.

## 3 Application of pooling procedures to wind droughts

### 3.1 Runs analysis

Runs analysis was applied to identify wind droughts from the GB-aggregated CF time series. Runs analysis originates from the theory of runs [12], initially developed to identify hydrological droughts. The method allows a sequence of events to be identified from the time series by assigning a value of one to the time steps where the data satisfy a specified threshold. In the present study, this becomes:

(2) $$\delta \left(t\right)=\left\{\begin{array}{cc} 1,\; & \text{if}CF\left(t\right)\leq \theta_{CF}\\ 0,\; & \text{if}CF\left(t\right)>\theta_{CF}, \end{array}\right)$$

where δ is a binary variable defined by whether the CF at time t is below a CF threshold $\theta_{CF}$ [29]. In this study, $\theta_{CF}$ values of 0.05, 0.10, and 0.20 were considered. Drought characteristics of interest are the drought duration d, defined as the length of time δ is consecutively equal to 1, and drought severity s, the energy deficit represented by the area between the CF time series and the CF threshold, as shown in Fig. 1.

### 3.2 Pooling procedures

High time resolution data series tend to identify many minor droughts, i.e., droughts of small duration and severity. The high frequency of these minor droughts may influence extreme value analysis conducted on the obtained drought series.

A second and more significant issue that can arise is the occurrence of mutually dependent droughts. These are defined as instances when a prolonged drought of long duration and high severity is interrupted by excess periods of relatively small duration and severity, thus dividing the single prolonged drought into several droughts. Fig. 1 provides an example where droughts 2 and 3 are separated by an excess period of relatively small duration $\gamma_{3}$ and severity $\sigma_{3}$. In the context of wind power production, such excess periods may occur due to short periods of higher wind speed localised to a limited number of wind farms with a high capacity within a longer period of generally low wind speeds. Mutually dependent droughts may therefore introduce errors into extreme value analysis by violating the assumption that the analysed events are independent.

Pooling procedures can be used to address the issues of minor and mutually dependent droughts as systematic processes that are performed alongside runs analysis to determine whether multiple drought events should be considered as separate events or as a single event. For example, droughts 2 and 3 in Fig. 1 may be considered to be mutually dependent and therefore pooled into one drought event. Therefore, pooling only allows the clustering of relatively minor droughts. However, it is important to note that while pooling can provide greater confidence that the resultant drought series comprises independent events, it does not fully guarantee such independence. Correlations between wind droughts which arise from mesoscale weather systems may still be present. Nonetheless, the application of extreme value analysis on a time series of pooled events is a generally accepted practice which provides a reasonable approximation [15].

**Fig. 4:**
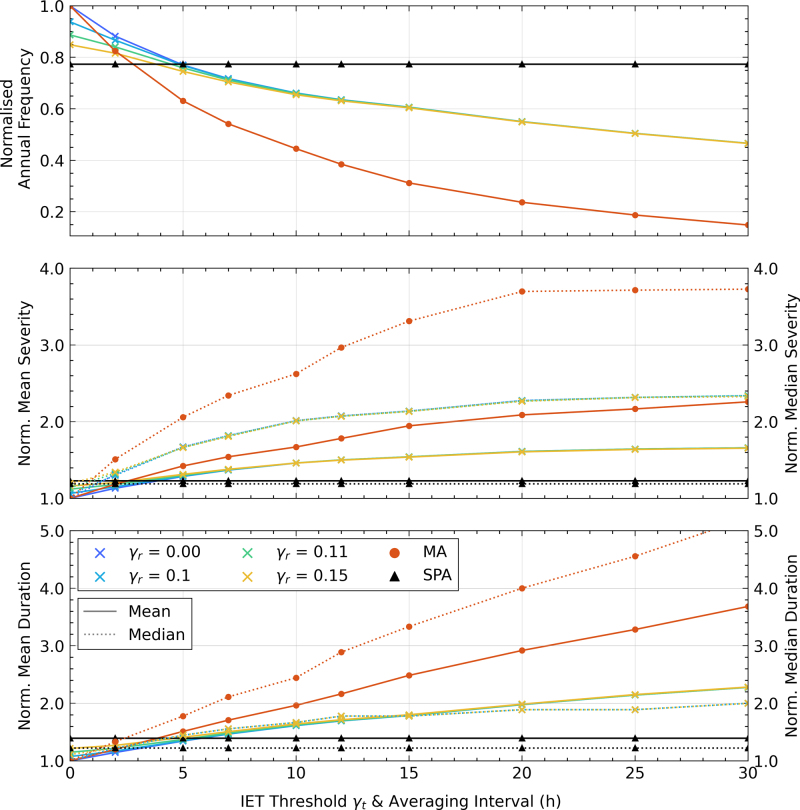
Drought metrics annual frequency (top), severity (middle), duration (bottom), normalised with respect to no applied pooling procedure, as functions of the inter-event threshold period $\gamma_{t}$ (IET) and averaging interval (MA). Inter-event ratios $0\leq \gamma_{r}\leq 0.1$ are shown for the modified IET method. The capacity factor threshold $\theta_{CF}=0.1$. Note that the SPA method is parameterless so its drought metrics are constant.

The duration of a pooled event $d_{pool}$ is defined as the sum of the durations of the pooled events including the excess period:

(3) $$d_{pool,i}=d_{i}+\overset{n}{\underset{j}{\sum }}\left(d_{j}+\gamma_{j}\right)$$

The pooled drought severity $s_{pool,i}$, is defined as the sum of pooled drought severity net of excess severity:

(4) $$s_{pool,i}=s_{i}+\overset{n}{\underset{j}{\sum }}\left(s_{j}-\sigma_{j}\right)$$

to reflect the net energy deficit presented to the electricity system.

Three pooling procedures have been applied to the CF time series in this study in order to evaluate drought duration and severity: Inter-Event Time (IET); Moving Average (MA); and the Sequent Peak Algorithm (SPA) method.

#### 3.2.1 Inter-event time method

The IET method, introduced by Zelenhasić and Salvai [9], considers drought events to be mutually dependent and thus pooled if the time between them is less than or equal to a predefined period $\gamma_{t}$. While the conventional IET method only considers absolute value of the inter-event time, we adapt the IET method to also consider drought events to be mutually dependent if the ratio of the excess period to the total duration of the drought events is less than or equal to a predefined ratio $\gamma_{r}$, i.e. $\gamma_{i}/\left(d_{i}+d_{i+1}\right)\leq \gamma_{r}$. This ensures that mutually dependent droughts are identified proportionately to their duration. For example, while drought events lasting 8 h separated by an excess period of 4 h may be considered as separate events, drought events lasting 100 h separated by an excess period of the same duration may be more suitably considered to parts of the same drought event. As such, IET pooling metrics in this study are defined by both the inter-event time $\gamma_{t}$ and the inter-event time ratio $\gamma_{r}$. IET pooling is evaluated iteratively, so droughts that are initially separate may eventually be pooled together on subsequent passes as a drought gets longer.

#### 3.2.2 Moving average method

The MA method [10] involves applying a moving filter with an n-hour interval to average the CF time series. As a result, short excess periods are filtered out and mutually dependent droughts are pooled. The degree of pooling is controlled by the length of the averaging interval n.

There are two caveats which arise from the smoothing of the CF time series. First, the pooled event may start after the first time step the series is below the threshold and may end before the last time step the series is below the threshold. Second, while it is standard to compute drought duration and severity from the modified time series, these will be different than those computed from the original CF time series due to averaging. Thus, here the moving-average-filtered time series was used only to identify the start and end of droughts, whereas the drought characteristics were computed with respect to the original time series.

**Fig. 5:**
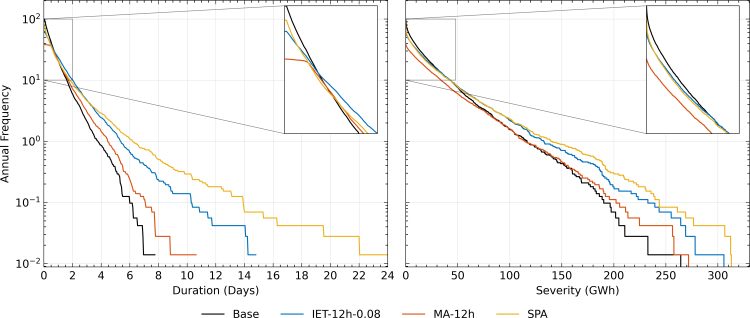
Frequency-duration (left) and frequency-severity (right) curves of wind droughts as determined by the IET (blue), MA (red), and SPA (yellow) pooling procedures presented on a logarithmic y-axis. The no-pooling baseline is presented in black. Droughts are defined with respect to a capacity factor threshold $\theta_{th}=0.1$.

#### 3.2.3 Sequent peak algorithm

The SPA method [14] is based on the energetic content of the underlying time series, and unlike the previous procedures, does not require user-specified parameters. A deficit time series w(t) is formulated by summing the hourly CF deficit (the difference between $\theta_{CF}$ and the CF time series below $\theta_{CF}$) and subtracting the excess CF (the difference between $\theta_{CF}$ and the CF time series above $\theta_{CF}$) until w(t) returns to zero. w(t) cannot be negative:

(5) $$w\left(t\right)=\left\{\begin{array}{cc} w\left(t-1\right)+\theta_{CF}-CF\left(t\right),\; & \text{if}w\left(t-1\right)+\theta_{CF}-CF\left(t\right)>0\\ 0,\; & \text{if}w\left(t-1\right)+\theta_{CF}-CF\left(t\right)\leq 0. \end{array}\right)$$

w(t) is therefore a time series of m uninterrupted periods of deficit. For each period i, the start of the drought event is defined when $w_{i}\left(t\right)>0$, and the end of the drought is defined as the time step where $w_{i}\left(t\right)$ reaches its maximum. The severity of the drought is computed based on the original CF time series per Eq. (4) by using the time steps obtained from $w_{i}\left(t\right)$. As such, drought events that are separated by an excess period whose excess energy is not sufficiently high to offset the severity of the drought events, i.e., w(t) remains above zero, will be considered to be mutually dependent and hence pooled. However, a well-recognised limitation of such an approach is that relatively minor droughts that follow a major drought are not identified because a local peak of w(t) is reached before the minor drought occurs. Therefore, a modified SPA pooling procedure is employed in this paper whereby w(t) is set to 0 at the time step that immediately succeeding a local peak in order to detect droughts that would otherwise be missed.

### 3.3 Sensitivity to pooling procedure

Fig. 4 shows the sensitivity of wind drought metrics to the parameters of each pooling procedure with a capacity factor threshold $\theta_{CF}=0.1$. Sensitivity is assessed relative to the annual frequency, duration, and severity, of all identified droughts normalised with respect to the base case where no pooling procedure is applied. As the SPA method is parameterless, its normalised metrics do not change as a function of inter-event threshold or moving-average interval.

Increasing the MA method averaging interval results in smoothing of the capacity factor time series which has the effect of eliminating shorter duration droughts. Consequently, the normalised annual frequency drops with an increase in averaging interval as fewer droughts are identified. Similarly, drought severity and duration increase as the shorter events are excluded and longer events are pooled together. The rate of increase of mean and median normalised severity reduces with averaging period due to the finite energy deficit of the pooled droughts (limited by $\theta_{CF}$), whereas duration continues to increase as the averaging interval tends to spread the droughts out in time.

For the IET method, the normalised drought metrics are presented as functions of the time threshold $\gamma_{t}$ and the time ratio threshold $\gamma_{r}$. All droughts are pooled into a single event for values of $\gamma_{r}$ exceeding 0.1, providing an upper limit.

Similar to the MA method, larger inter-event threshold times $\gamma_{t}$ result in more droughts being pooled together, hence the normalised annual frequency reduces, albeit not as dramatically as the MA method where averaging of the CF time series can remove some shorter droughts. Similarly, drought duration and severity increase with $\gamma_{t}$ as more events are pooled together although preserving shorter droughts means that the mean and median statistics do not change as significantly as for the MA method.

To enable cross-comparison between the MA, IET, and SPA methods, intervals have been chosen that balance pooling droughts together and minimising the modification of the underlying time series through averaging [15]. For the MA method, an averaging interval of 12 h was adopted, and for the IET method, $\gamma_{t}=12$ h and $\gamma_{r}=0.08$ were selected.

### 3.4 Frequency, duration, and severity of drought events

Annualised frequency-duration and frequency-severity curves for $\theta_{CF}=0.1$ are presented in Fig. 5 alongside summary statistics in Table 1 to illustrate the differences in the wind droughts identified by the different pooling procedures. The shallower gradients of their frequency-duration and -severity curves with respect to the baseline shows that pooling allows longer events to be identified while also filtering out minor droughts. This is reflected in the decreased annual frequency of droughts and the increase in mean, median, and maximum duration and severity.

The MA method results in the largest decrease in the annual frequency as droughts of less than the averaging period are filtered out, seen as the plateau in the MA frequency-duration curve. There is an associated large increase in mean and median duration and severity as relatively small droughts are neglected. While the maximum drought duration increases from the baseline case, it is not as significant as for the IET or SPA methods.

Compared to the MA method, the IET frequency-duration and -severity curves display a higher frequency of longer duration and more severe droughts. Consequently, the maximum drought duration and severity with IET method is greater than identified by the MA method. However, the changes in mean and median drought duration and severity from the no-pooling baseline case are smaller than those with the MA method as the IET method also preserves the frequent short duration and low severity droughts.

The SPA method demonstrates the greatest increase in the frequency of long and severe droughts compared to the no-pooling baseline. While the maximum severity of SPA-identified droughts is comparable to the other methods, the maximum drought duration reaches up to 616 h, more than a threefold increase compared to the baseline case. Hence, it is evident that the SPA’s distinct approach that considers the CF time series’ energetic content leads to the identification of much longer and more severe droughts.

However, compared to the baseline, the SPA-identified drought series demonstrates the smallest decrease in annual frequency as well as the lowest increase in mean and median duration and severity. This is because the SPA method preserves the most minor droughts out of all three pooling procedures. While the SPA is able to pool many droughts over a long period, it does not pool minor droughts that precede a major one, hence more minor droughts are preserved in the pooled series.

**Table 1:** Drought statistics for IET, MA, and SPA pooling procedures with a capacity factor threshold $\theta_{th}=0.1$.

Pooling procedure	Annual frequency	Duration (h)			Severity (GWh)		
		Mean	Median	Max	Mean	Median	Max
Base	99.6	15.5	9	187	14.8	5.0	278.5
IET-12h-0.08	63.1	26.4	16	356	22.2	10.5	314.7
MA-12h	38.3	33.5	26	256	26.3	15.0	304.0
SPA	77.0	21.6	11	616	18.2	6.0	314.8

**Table 2:** Extreme value series analysed in this study.

	AMS	PDS
Duration	AMS Duration ($D_{AMS}$)	PDS Duration ($D_{PDS}$)
Severity	AMS Severity ($S_{AMS}$)	PDS Severity ($S_{PDS}$)

## 4 Extreme value analysis: a 72-year case study of wind droughts in Great Britain

### 4.1 Extreme value models

**Fig. 6:**
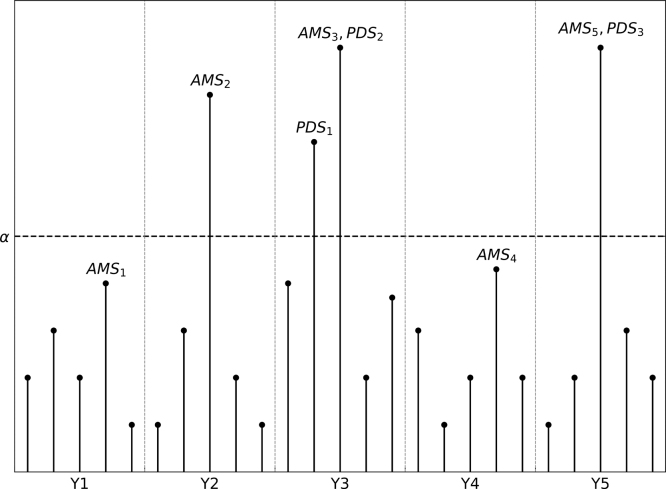
An illustration of a hypothetical discrete wind drought series where each stem corresponds to a drought event of a certain magnitude and grey dashed lines mark the end of each year. Suppose such a series is five years long and contains five events each year. Events with the annually highest magnitude are included in the AMS series, while those whose magnitudes are greater than a truncation level α are included in the PDS.

Two commonly-used approaches to define extreme values of severity and duration from the drought time series were considered: the Annual Maximum Series (AMS) model and the Partial Duration Series (PDS) model. Their differences are summarised below and in Fig. 6.

In the AMS framework, the event with the highest magnitude within each year is extracted for extreme value analysis. Events spanning multiple years are assigned to the year they begin. While the main advantage of using the AMS model is its simplicity, there are a number of caveats. First, if the chosen $\theta_{CF}$ is too low so that there are years without any drought events, the extreme event sample size may be small, impacting on the derived statistics. The CF threshold values used in this study are sufficiently high to avoid such occurrences. Second, extracting the highest magnitude event of each year does not guarantee that the extracted sample contains events with the highest magnitudes from the population, i.e., two events with the highest magnitudes in the population may occur in the same year and hence, the event with the second highest magnitude is omitted from the sample. In this study, the AMS extreme value series contains 72 events.

The PDS model includes drought events that they exceed a certain truncation level α. The truncation level α is different from the CF threshold $\theta_{CF}$ as α is defined with respect to the discrete wind drought duration and severity series, not the CF series. The main advantage of the PDS model is that all events with the highest magnitude are included, providing a more robust definition of the extreme events than the AMS model. However, it is sensitive to the truncation level α. While systematic methods exist to select α, in practice, this process is determined based on subjective judgement and often relies on values adopted by existing studies [15]. In this study, the 0.95 quantile for each drought variable was selected as the truncation level.

In this study, both models were used to define the extreme value region for both wind drought duration and severity series, and the results from both models are compared. Hence, for each $\theta_{CF}$, four extreme value series were analysed as per Table 2.

#### 4.1.1 Distribution fitting and evaluation

Theoretical extreme value distributions can provide a means to robustly extrapolate from a limited extreme value sample and describe the sample’s statistical properties. Five distributions commonly used to represent extreme hydrological drought events were investigated: Lognormal, Generalised Extreme Value (GEV), Pearson Type III (Pearson-III), Generalised Pareto (Gen. Pareto), and Generalised Logistic (Gen. Logistic) distributions [10], [15]. The GEV distribution conventionally has three parameters, and other distributions were also considered in their three-parameter form where the third parameter is the location parameter. The distributions were fitted to each of the four extreme value series using maximum likelihood estimation.

The suitability of the distributions was evaluated based on the Cramér–von Mises (CvM) test and Akaike Information Criterion (AIC). The distribution which passes the CvM test and achieves the highest AIC was chosen as the representative theoretical distribution for a given extreme value series. Additionally, an L-moment ratios diagram was used to compare the L-skewness and L-kurtosis to the L-moment ratio loci of each candidate theoretical distribution.

#### 4.1.2 Return periods and confidence intervals

Following Shiau [11], the return period for a univariate extreme value series can be calculated assuming that the events are independently and identically distributed. Defining X to be an independent drought event of magnitude x, L the time period between any two successive events, and T the time period between two successive events with magnitudes greater than or equal to x, i.e., the recurrence interval. The recurrence interval for event X≥x is then:

(6) $$E\left[T\right]=\frac{E\left[L\right]}{1-F\left(x\right)},$$

where E[L] is the expected inter-arrival time between successive extreme events and F(x) is the cumulative density function of the drought variable. For the AMS series, since events are sampled annually, E[L]=1, whereas for the PDS series, E[L] is the mean inter-arrival rate in years.

The return period of each extreme value series was calculated according to Eq. (6), using the fitted distributions selected as described in Section 4.1.1. Next, the associated uncertainty was obtained using non-parametric bootstrapping. The underlying extreme value series was randomly sampled with replacement so that the bootstrapped sample size matched that of the extreme value series. The selected theoretical distribution was fitted to the bootstrapped sample, and return period was calculated as per Eq. (6). This process was repeated to generate 500 bootstrapped samples and at each x, the 95% confidence interval is presented.

**Table 3:** Results from the CvM test and AIC presented for AMS duration ($D_{AMS}$), PDS duration ($D_{PDS}$), AMS severity ($S_{AMS}$), and PDS severity ($S_{PDS}$) at $\theta_{CF}=\left(0.05,0.10,0.20\right)$. Droughts were pooled using the SPA procedure. The theoretical distribution for each extreme value series whose AIC value is highlighted in bold was selected as the representative distribution.

$\theta_{CF}$	Distribution	$D_{AMS}$		$D_{PDS}$		$S_{AMS}$		$S_{PDS}$	
		p-value	AIC	p-value	AIC	p-value	AIC	p-value	AIC
**0.05**	**Lognormal**	0.814	711.2	0.622	1626.4	0.368	605.7	0.344	1387.8
	**GEV**	0.708	712.9	0.325	1641.2	0.338	606.5	0.260	1404.2
	**Pearson-III**	0.947	**709.3**	0.569	**1524.9**	0.444	**604.8**	0.849	**1358.3**
	**Gen. Pareto**	0.501	710.7	0.437	1632.7	0.112	611.5	0.647	1396.6
	**Gen. Logistic**	0.638	715.4	0.001	1730.7	0.362	606.9	0.010	1454.1

**0.1**	**Lognormal**	0.983	**835.0**	0.537	2770.8	0.860	787.9	0.386	2733.8
	**GEV**	0.920	836.4	0.477	2785.8	0.000	1051.1	0.219	2755.3
	**Pearson-III**	0.966	835.4	0.244	2796.6	0.869	**787.0**	0.683	**2710.2**
	**Gen. Pareto**	0.398	839.9	0.675	**2765.2**	0.259	789.9	0.637	2723.4
	**Gen. Logistic**	0.555	844.9	0.001	2898.9	0.784	788.6	0.002	2829.1

**0.2**	**Lognormal**	0.942	1081.0	0.864	3674.0	0.000	1273.7	0.676	3767.7
	**GEV**	0.835	1083.9	0.738	3689.4	0.000	1325.3	0.482	3784.7
	**Pearson-III**	0.952	**1080.3**	0.002	3661.9	0.752	**1056.7**	0.113	**3745.0**
	**Gen. Pareto**	0.815	1081.1	0.728	**3672.6**	0.001	1070.5	0.909	3753.7
	**Gen. Logistic**	0.282	1098.4	0.000	3949.0	0.460	1063.1	0.000	3911.7

### 4.2 Extreme value distribution evaluation

The SPA method was chosen as the drought pooling procedure due to the favourable performance noted in Section 3.3. Hence, $D_{AMS}$, $D_{PDS}$, $S_{AMS}$, and $S_{PDS}$ are modelled on the drought events identified by the SPA procedure. Capacity factor thresholds of $\theta_{CF}=\left(0.05,0.10,0.20\right)$ are investigated.

**Fig. 7:**
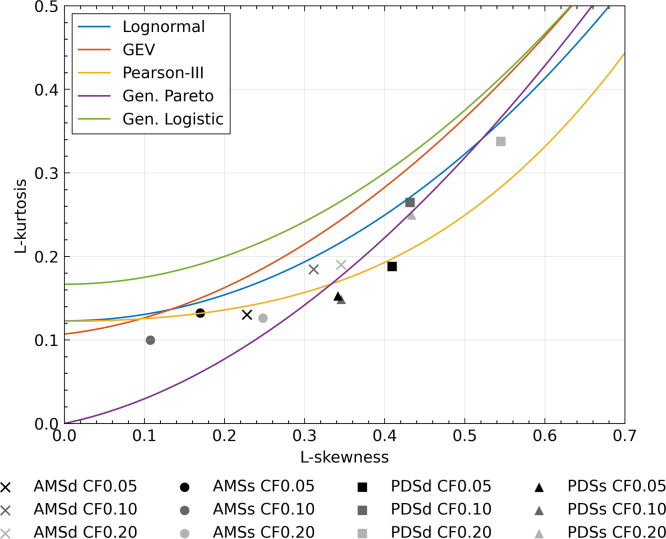
L-skewness vs. L-kurtosis of candidate theoretical extreme value distributions compared to the AMS and PDS duration and severity series for three capacity factor thresholds $\theta_{CF}=\left(0.05,0.10,0.20\right)$.

Fig. 7 illustrates the loci of L-skewness/L-kurtosis for all candidate distributions as well as for the extreme value series data. The upwards curvature of the L-moment ratios reflects the heavy tails of the extreme value distributions. Higher values of $\theta_{CF}$ tend to result in heavier-tailed and more asymmetric distributions than for lower values of $\theta_{CF}$. Similarly, the PDS data tend to be heavier tailed and more asymmetric than their AMS counterparts for the same $\theta_{CF}$. This is driven by the greater number of extreme events identified for higher $\theta_{CF}$ or with the PDS method.

It is observed in Fig. 7 that the extreme value series are fairly well modelled by the Pearson-III distribution for lower $\theta_{CF}$ values, whereas the Gen. Pareto distribution and lognormal distributions better model the data at higher $\theta_{CF}$. Additionally, the Gen. Pareto distribution models all the PDS series well, consistent with extreme value theory which posits that excess values over a certain threshold are Gen. Pareto distributed. The Gen. Logistic distribution does not appear to model the extreme value series well.

These findings are supported by the CvM test and AIC as presented in Table 3. For $\theta_{CF}=0.05$, the CvM test failed to reject the null hypothesis that the extreme value series are drawn from any of the candidate distributions except for the Gen. Logistic distribution for the $D_{PDS}$ case. The Pearson-III distribution achieved the lowest CvM test statistic as well as the lowest AIC score across all extreme value series. This further implies that the Pearson-III distribution adequately captures extreme value series for lower values of $\theta_{CF}$, with the GEV and Lognormal distributions as suitable alternatives.

For the extreme value series corresponding to $\theta_{CF}=0.10$, the CvM test and AIC results are less conclusive. While the Pearson-III distribution was chosen for the severity cases $S_{AMS}$ and $S_{PDS}$, the Lognormal and Gen. Pareto distributions were most suitable distributions for the duration cases $D_{AMS}$ and $D_{PDS}$ respectively. In most cases, these distributions offer suitable alternatives to each other as well.

For $\theta_{CF}=0.20$, the Pearson-III distribution remains the best-fit distribution for all series except for $D_{PDS}$, which is best represented by the Gen. Pareto distribution. The Lognormal and Gen. Pareto distributions are suitable alternatives for the $D_{AMS}$ and $S_{PDS}$ series, while for $S_{AMS}$, the Gen. Logistic distribution is the second best-fit distribution.

In general, the Pearson-III distribution performs best at modelling most of the extreme value series, with the Lognormal and Gen. Pareto distributions offering adequate alternatives, especially for PDS series for the Gen. Pareto distribution.

### 4.3 Return periods and confidence intervals

**Fig. 8:**
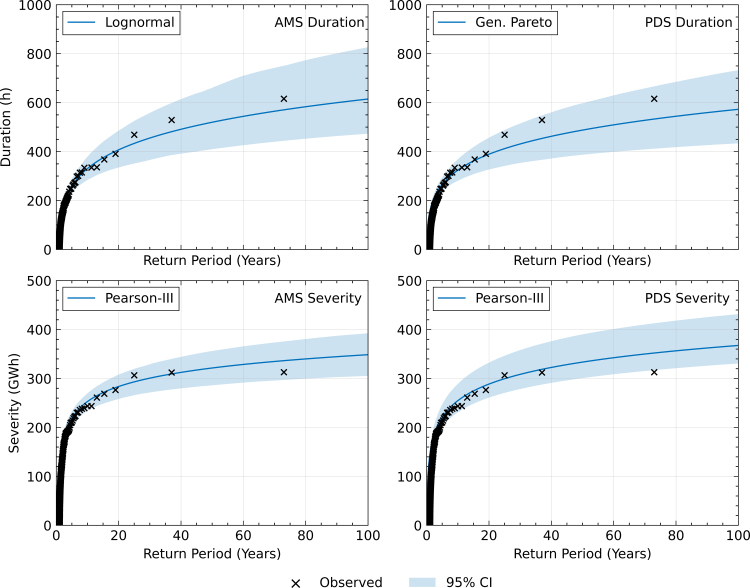
Return levels and the 95% confidence interval plotted against return periods calculated from the best-fit theoretical distribution for $D_{AMS}$, $D_{PDS}$, $S_{AMS}$, and $S_{PDS}$ corresponding to $\theta_{CF}=0.10$. Black crosses show return periods empirically computed from the observed extreme drought series.

Using the best-fit theoretical distribution for each $\theta_{CF}=0.10$ extreme value series, the corresponding return period curves and their 95% confidence intervals, as well as empirically computed return periods are presented in Fig. 8. The return levels for selected periods for all extreme values series at $\theta_{CF}=\left(0.05,0.10,0.20\right)$ are summarised in Table 4. All curves are concave, reflecting a diminishing probability of more extreme, rarer events. The return period curve shows good agreement with empirically calculated return periods up to a return period of 20 years. The uncertainty around the rarest events is captured by the widening confidence interval, increasing from ±12% for a 5 year return period compared to ±22% at 50 years. This is due to rarity and thus limited sample size of the most extreme events. Nevertheless, the observed return periods are within the 95% confidence intervals at these higher return periods.

Furthermore, the return levels associated with the AMS and the PDS approaches for drought severity and duration at a given return period are in reasonable agreement, differing by 15% and 4% respectively. This suggests that both approaches are able to capture the characteristics of extreme events, providing confidence in the reliability and robustness of the estimated return periods and levels.

A wind drought defined by $\theta_{CF}=0.10$ lasting approximately 1 week is expected to occur once every 2 years, and once every 10 years for a circa 2 week drought. The longest wind drought in the 72-year record is 616 h, approximately 26 days, has a return period of 100 years. As the droughts are identified by the SPA procedure, it is important to note that the return duration does not necessarily indicate that the CF time series never exceeds $\theta_{CF}$ during the period, but rather signifies that the energetic content of the CF time series is in deficit with respect to the $\theta_{CF}$ throughout the period. An SPA-defined wind drought lasting at least 1 week occurs 53 times over the 72 year study period whereas a 1 week period where the CF time series never exceeds $\theta_{CF}$ occurs only 3 times over the same 72-year period.

Drought severity refers to the energy deficit with respect to constant production at the level of $\theta_{CF}$. For example, relative to $\theta_{CF}=0.10$ using the $S_{AMS}$ estimates, a wind drought with a deficit of 165 GWh is expected to occur once every 2 years, while the most severe wind drought in the 72 year record of 315 GWh is expected to occur slightly under once every 50 years. Similar to wind drought duration, the SPA method identifies some droughts with relatively modest severity due to pooling mutually dependent events together and thus also capturing intermediate periods where the instantaneous $CF>\theta_{CF}$, limiting drought severity. Within the 72 years of data, an SPA-defined wind drought corresponding to $\theta_{CF}=0.10$ of at least 165 GWh occurs 53 times.

**Table 4:** Return periods and confidence intervals for the AMS and PDS series of SPA-identified drought duration and severity at different CF thresholds.

$\theta_{CF}$	E[T] (y)	$D_{AMS}\left(h\right)$			$D_{PDS}\left(h\right)$			$S_{AMS}\left(GWh\right)$			$S_{PDS}\left(GWh\right)$		
		Fit	0.025	0.975	Fit	0.025	0.975	Fit	0.025	0.975	Fit	0.025	0.975
**0.05**	**2**	79	62	88	89	83	96	39	35	43	44	39	51
	**5**	116	101	133	127	111	142	55	50	62	56	48	68
	**10**	142	122	163	167	139	194	66	58	76	65	56	80
	**50**	198	160	245	306	227	391	89	73	108	87	72	110
	**100**	221	161	281	391	278	520	98	79	121	96	79	123

**0.1**	**2**	173	154	195	198	183	214	165	149	181	176	157	199
	**5**	260	223	300	266	235	298	219	201	237	221	194	258
	**10**	331	273	391	324	277	379	253	227	274	255	223	303
	**50**	520	391	683	488	373	644	321	272	370	334	290	410
	**100**	615	445	854	573	418	809	349	291	409	368	319	457

**0.2**	**2**	699	421	819	684	605	777	870	736	964	926	734	1105
	**5**	1244	849	1615	1127	938	1365	1271	1099	1450	1224	948	1493
	**10**	1643	1190	2284	1616	1261	2093	1544	1331	1794	1452	1111	1805
	**50**	2551	1888	3766	3605	2394	5582	2137	1793	2641	1987	1483	2564
	**100**	2938	2164	4439	5050	3124	8480	2383	1974	3018	2218	1642	2889

## 5 Discussion

### 5.1 Implications for the electricity system

**Fig. 9:**
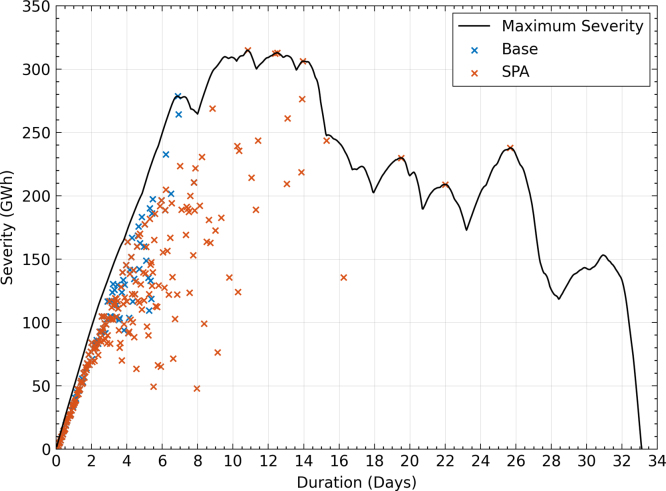
Maximum event severity adapted from Ruhnau and Qvist’s energy storage model. Black curve shows the maximum drought severity for a given overlapping time window. Points refer to the maximum drought severity for each event duration as identified by a simple runs analysis and by the SPA method.

To demonstrate the relationship between the severity of wind droughts and energy storage requirements, a basic energy storage model adapted from Ruhnau and Qvist [30] was employed. In this model, severity is calculated based on a time window of length L applied in an overlapping manner across the time series. The maximum severity for each L is then recorded. The model was applied on the GB-aggregated time series with respect to a capacity factor threshold of 0.1 and the results are plotted in Fig. 9, against the most severe droughts at each duration as identified by SPA and the baseline runs analysis case. First, it can be observed more clearly that the relationship between extreme drought duration and severity is non-linear i.e., the most severe drought is not necessarily the longest. In this case, the peak of the curve, which, by Ruhnau and Qvist’s [30] definition, corresponds to the energy storage requirement at 0.1 CF threshold, has a severity of 314.8 GWh. But more importantly, this peak, along with other prominent peaks, are also captured by the SPA method, but not by a simple runs analysis.

This result shows that using pooling procedures to assess wind droughts provides a novel perspective on estimates of the risks of droughts for the electricity system as well as an understanding on their implications for energy storage. This is because in this framework, prolonged wind droughts are considered not as periods where the *wind power* never exceeds a specified threshold, but rather that *wind energy* generation falls below a threshold throughout the drought. Hence, the use of SPA procedures offers a time-series-based tool to estimate energy storage requirements of an electricity system from a resource perspective. We note that the SPA analysis identifies similar maximum severity droughts as the energy storage model of Heide et al.’s [31] model, which was developed in the context of energy storage modelling from an electricity system perspective. However, identification of less severe droughts with SPA analysis allows statistical models of wind droughts to be developed as discussed in Section 4.

Return periods estimated from this definition of wind droughts suggest that the likelihood of prolonged periods of low wind energy may be higher than previously understood. While the severity of droughts, defined in terms of the energy deficit, do not necessarily increase as significantly as their duration due to pooling, these wind droughts still pose a challenge to the resilience of the electricity system. Additionally, we note that the current (2022) GB energy storage capacity was estimated to be approximately 115 GWh [32]. The estimated return period for severity suggests that GB’s current energy storage would satisfy events with a return period of 1.17 years in order to maintain a net energetic output corresponding to CF=0.10.

As with findings from Cannon et al. [3] and Potisomporn and Vogel [4], the majority of the identified wind droughts occur in spring and summer, with the longest events also occurring in these seasons. Electricity demand in European countries however is generally higher in winter [33], so a wind drought could have greater impact in winter even if it is not as severe as others. To gauge the impacts that these wind droughts have on the electricity system, the drought pooling and extreme value analysis outlined in this paper was employed on a daily time series of electricity demand net of wind energy generation (demand-net-wind). Further details are available in the Supplementary Information.

Droughts are defined to correspond to meeting or exceeding the 0.9 quantile of demand-net-wind, with the resulting return periods presented in Table 5. The reader is reminded that SPA pooling means that droughts are not defined as periods where the 0.9 quantile threshold is exceeded continuously, but rather as periods where there is an energy deficit with respect to this threshold, ending only when the deficit peaks.

Unlike droughts defined only on wind generation, over 80% of the events identified from the demand-net-wind time series occur in winter and autumn, and none occur in summer. A demand-net-wind drought that lasts approximately 10 days is estimated to occur once every 2 years while a wind drought that lasts approximately one month is expected to occur once every 10 years. The maximum event duration identified in the dataset is 68 days and is expected to occur once every approximately 80 years. An event with a severity of 755 GWh, or 6.5 times the current energetic capacity of the UK wind energy storage, is estimated to occur once every 2 years while the most severe event that was observed in the 72 year record, with a severity of 4.8 TWh, is expected to occur once every 150 years. At present, a mix of dispatchable (generally fossil-fuelled) and baseload generation is used to satisfy the residual demand net of wind generation. However as the generation portfolio changes in future there may be a greater need for interconnection to neighbouring countries and energy storage technologies to satisfy the mismatch between wind generation and energy demand.

**Table 5:** Return periods and confidence intervals for the AMS and PDS series of SPA-identified droughts duration and severity from demand-net-wind.

E[T] (y)		$D_{AMS}$	$D_{PDS}$	$S_{AMS}$	$S_{PDS}$
		(days)	(days)	(GWh)	(GWh)
**2**	**Fit**	10.7	10.2	755	547
	**0.025**	9.2	9.7	706	525
	**0.975**	12.8	11.3	875	584

**5**	**Fit**	19.2	18.7	1325	1087
	**0.025**	16.0	16.2	1261	945
	**0.975**	23.2	21.9	1546	1292

**10**	**Fit**	27.3	26.5	1783	1520
	**0.025**	21.7	21.0	1689	1253
	**0.975**	35.3	31.8	2115	1814

**50**	**Fit**	57.4	52.3	3014	2744
	**0.025**	37.4	34.1	2785	1921
	**0.975**	92.6	63.1	3691	3715

**100**	**Fit**	77.6	67.6	3629	3400
	**0.025**	44.8	40.9	3250	2119
	**0.975**	139.1	81.9	4528	5001

### 5.2 Model limitations

The extreme value analysis conducted in this study provides novel insights into the frequency of wind droughts. Using pooling procedures strengthens the assumption of event independence and an Augmented Dickey–Fuller test validated that the CF time series can be considered to be stationary. However, there are also limitations and uncertainties associated with the analysis. The appropriateness of drought pooling, the choice of pooling procedure, and the identification of extreme events through annual maximum series or partial duration series are all intertwined with the structure of the electricity system and how it is affected by wind droughts.

The GB-aggregated CF time series was synthesised from ERA5 reanalysis data. The ability of macro-scale reanalysis models to reproduce periods of low wind energy has been reported previously [21]; while small, short period events tend to be underestimated, it can reproduce longer-period events. Extended datasets such as the 72 years offered by ERA5 are important for extreme value analysis, although this is still limited when estimating confidence intervals for return periods longer than 20 years, as shown in Fig. 8. Bootstrapping was adopted to address this limitation, but other methods such as the use of simulated and ensemble data could also be used to increase the sample size.

The extreme value analysis in this study was conducted separately on drought duration and severity. However, a wind drought is characterised by both of these variables simultaneously which are necessarily related. Thus, the traditional approach of using distinct univariate distributions to describe duration and severity may overlook the interdependence of the variables. An area of future work is to investigate bivariate distributions and joint return periods (see e.g. [11]) to better characterise prolonged wind droughts.

## 6 Conclusions

Wind droughts, prolonged periods of low wind power generation, represent a major challenge in the decarbonisation of the GB electricity system. Using the 72 year ERA5 reanalysis dataset, this study has leveraged the similarities between wind and hydrological droughts to apply well-established statistical techniques and methodologies to conduct an extreme value analysis of wind droughts in GB.

Pooling procedures in conjunction with runs analysis, are commonly employed hydrological techniques to identify and group drought periods. Application of the IET, MA, and SPA methods to the GB-aggregated capacity factor time series, allowed the identification of longer wind drought periods, during which wind power generation may not necessarily be below a given threshold at all times, but the energy deficit below a threshold may nevertheless have significant impacts on the electricity system. This approach to examining wind droughts may be particularly relevant to the evaluating the role and need for energy storage technologies.

While each pooling procedure has strengths and weaknesses, SPA was found to identify the longest events whilst also preserving more frequent short events, and does not require tuning of model parameters such as averaging periods. Using the SPA pooling procedure, it was found that wind droughts corresponding to a $\theta_{CF}=0.10$ could persist for a duration of up to 26 days.

Extreme wind droughts were identified using the AMS and PDS methods, with the Pearson-III distribution modelling the extreme droughts well across a number of capacity factor thresholds. The Lognormal distribution was also found to work well for some extreme value series. Additionally, the Generalised Pareto distribution was particularly suitable for modelling PDS-extracted extreme value data, consistent with extreme value theory.

In the case study conducted on GB’s wind farm fleet, a wind drought defined below $\theta_{CF}=0.10$ using SPA pooling with a duration up to one week could be expected to occur as frequently as once every 2 years, while the longest drought in the 72 year record, which persisted for 26 days, is expected to occur once every 100 years. A similar extreme value analysis was also carried out on the demand-net-wind time series to incorporate electricity demand into the analysis. It was found that the current estimated total energetic capacity of GB energy storage is equivalent to sustained periods where demand-net-wind is above 0.9 quantile with a one year return period.

These results suggest that the March 2021 wind drought, with a duration of 11 days and an energetic deficit of 1 TWh with respect to demand-net-wind, was not a particularly rare event. In fact, a wind drought with comparable duration and severity occurred in December of the same year. In light of this, it is clear that the growing reliance on wind energy necessitates the development of strategies to mitigate the impacts of these wind droughts on the electricity system.

## CRediT authorship contribution statement

**Panit Potisomporn:** Methodology, Data curation, Software, Writing – original draft, Validation, Writing – review & editing, Formal analysis, Visualization, Investigation. **Thomas A.A. Adcock:** Conceptualization, Writing – review & editing, Methodology, Supervision, Resources. **Christopher R. Vogel:** Conceptualization, Writing – review & editing, Methodology, Supervision, Resources.

## Declaration of competing interest

The authors declare that they have no known competing financial interests or personal relationships that could have appeared to influence the work reported in this paper.

## Appendix A Supplementary data

MMC S1 GB-aggregated capacity factor and electricity demand models can be found in supplementary material.
