# Supplementary material for: Extreme value analysis of wind droughts in Great Britain
Source: Renew Energy. 2024 Feb;221:None. doi: 10.1016/j.renene.2023.119847 (PMC13333602; doi:10.1016/j.renene.2023.119847)
Supplement: MMC S1 — GB-aggregated capacity factor and electricity demand models can be found in supplementary material. [file mmc1.pdf]

Extreme Value Analysis of Wind Droughts in Great Britain - Supplementary Information

Panit Potisomporn[a], Thomas A.A. Adcock[a], Christopher R. Vogel[a]

[a] *Department of Engineering Science, University of Oxford, Parks Road, Oxford, OX1 3PJ, United Kingdom*

1. GB-aggregated CF model

The GB-aggregated capacity factor model used in this study can be summarised as follows. First, ERA5 10-m wind speed was evaluated and bias-corrected. Second, the corrected wind speed was extrapolated to hub height. Third, the hub height wind speed was converted to power output at a farm level. Last, power outputs from all wind farms were aggregated to obtain the GB-aggregated power output, which was then converted to capacity factor. Note that while ERA5 100-m wind speed is available, the methodology in this study is based on the 10-m wind speed due to a significantly higher availability of observed wind speed data at 10 m, against which ERA5 10-m wind speed was evaluated. Therefore, this approach provided a more thorough and robust understanding of the associated uncertainties.

1.1. Bias correction of ERA5

Following Potisomporn et al.'s [1] evaluation of ERA5 10-m wind speed, which was carried out with respect to 205 observation stations around the UK from the period 1997-2021, the values of bias reported were used in this study to inform the bias correction process of such data. Such a correction was performed by means of mean-variance scaling, which involves removing biases in mean wind speed and wind speed standard deviation from the spatially interpolated ERA5 10-m wind speed at each wind farm. The process to obtain the bias-corrected wind speed $u^*_{ERA}(t)$ from the uncorrected ERA wind speed $u_{ERA}(t)$ is best summarised by:

$$u^*_{ERA}(t) = [u_{ERA}(t) - \mu_{ERA}] \cdot \frac{\sigma_e}{\sigma_{ERA}} + \mu_e. \quad (1)$$

where μ_{ERA} and σ_{ERA} are the mean and standard deviation of the uncorrected wind speed respectively, while μ_e and σ_e are the reported biases in mean wind speed and standard deviation respectively.

1.2. Extrapolation of wind speed to hub height

In this study, a *Multi-Layer Perceptron Regression* (MLPR) model, a feed-forward subset of the Deep Neural Network algorithm coupled was used to vertically extrapolate 10-m wind speed to 100 m at each wind farm. Multi-layer Perceptron Regression comprises of neurons that act as logistic units, summing up weighted input signals and passing the resultant signal through an activation function [**?**]. These neurons are arranged into a network that always consists of an input layer that represents the input features, followed by a variable number of hidden layers of neurons before concluding at an output layer where the desired format of the signal is returned.

The inputs of the model are all available within the ERA5 model and ERA5 grid points in the UK domain without any spatial interpolation at regular spatial intervals and hourly time steps. Features included can be classified into three categories ,namely physical, geographical, and temporal. The selection of physical features was largely influenced by Monin-Obukhov's similarity theory so that the model reflects the physical reality of vertical extrapolation of wind speed and so, include 10 m wind speed, 10 m wind direction, friction velocity, 2 m temperature, surface roughness, and boundary layer height. Geographical features include distance to coast and elevation. Lastly, temporal features include the time of day and day of the year to capture the seasonal and diurnal variations. The model was trained on ERA5 100 m wind speed and thus includes ten inputs and one output. The summary of these features and their correlations (Pearson R) are presented in Figure 1.

Using these features, the MLPR model was trained on one out of every eight ERA5 grid points, sampled from randomly selected years, representing approximate 14 million data points. The trained Neural Network model was validated against ERA5 100 m wind speed data drawn from 169 random locations that were not included in the training of the model, sampled from a randomly selected two-year period not included in the training, thus representing 25% of the training set size. The results presented in Figure 2 show MLPR's superiority in performance over a simple Log-Law approach. First, the mean RMSE of these test locations was reduced significantly from 0.91 m/s to 0.49 m/s when Log-Law extrapolation was replaced by the Neural Network approach. While the averages of mean error, standard deviation error, scale parameter error, and shape parameter error do not differ significantly, the ranges of these errors are much smaller under the Neural Network approach. For example, the ranges of mean error and standard deviation

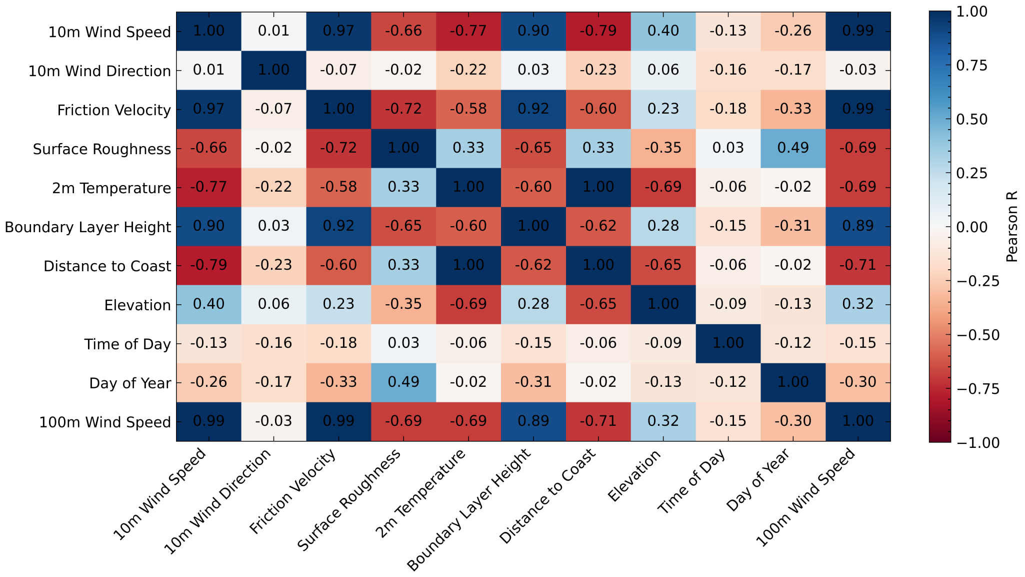

Figure 1: Matrix of Pearson R correlation between each feature used in training the model as well as the target variable.

error were reduced from 1.29 m/s to 0.72 m/s and from 0.85 m/s to 0.28 m/s respectively. Furthermore, mean Pearson R values also increased moderately when MPLR was used to extrapolate wind speed to 100 m.

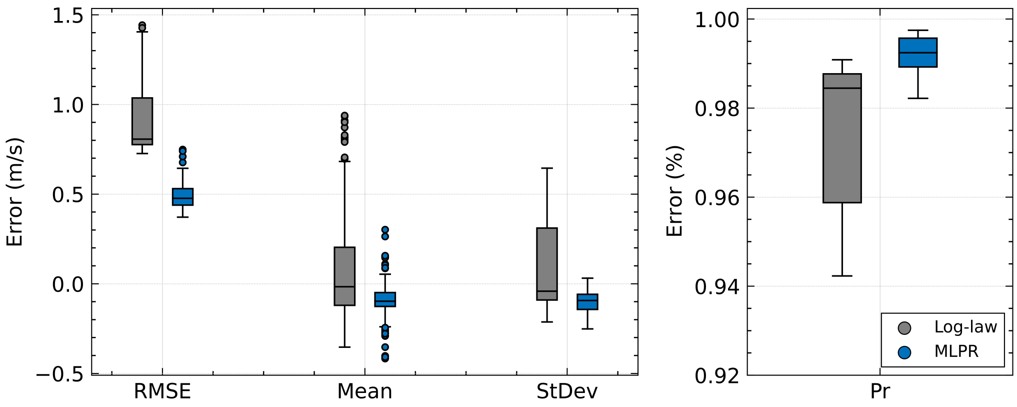

Figure 2: Error metrics from 169 randomly selected ERA5 test grid boxes.

1.3. Wind farm power curve modelling

In this study, a *wind farm power curve model* was developed to model the relationship between wind speed and power output at a farm level, as opposed to employing a manufacturer's power curve. The process to construct such a power curve can be summarised as follows. First, following the approach taken by [2], each wind farm was assigned based on the farm's mean wind speed, and was assigned one of the following three representative manufacturer's turbine power curves based on their International Electrotechnical Commission (IEC) classification: Siemens SWT-3.6-107 (Class I), GE General Electric GE 1.5SL (Class II), and ENO Energy 100 2.2 MW (Class III). Note that this is a classification system published by the IEC [3], which captures the variation of the shape of power curves with the wind speed at which they are designed to operate and hence, provides greater specificity to the model. Second, graphical quantities of the representative manufacturer's power curve were used to formulate a five-parameter logistic (5PL) curve, whose formula is given by:

$$P_{5PL} = f(u, \theta) = \frac{D + (A - D)}{(1 + (u/C)^B)^G} \tag{2}$$

to provide the model with a parametric, continuous transfer function. That the 5PL curve was used to represent the shape of the manufacturer's curve is based on findings that such a function shows substantial accuracy in estimating power output from a turbine when validated against measured power output data [4]. Note that due to limited data availability close to the cut-off speed, this section was fitted by inspection, as has been commonly done in parametric power curve modelling [**?**]. Third, to simulate farm-level effects, namely wake losses, measured wind farm power output data from Penmanshiel and Kelmarsh wind farms [5] were used to inform empirical calibrations of the 5PL curve to achieve the desired loss. Lastly, wind farm energetic availabilities obtained from Cevasco et al. [6] of 99.2% and 88.1% were applied to onshore and offshore wind farms respectively to take farm downtime into consideration. Note that these steps were carried out by using normalised power curve so that the output of this process is presented as wind farm capacity factor.

The resultant Class III power curves for both onshore and offshore are compared to the original manufacturer's power curve and presented in Figure 3 as an example. Two features of such curves are of interest. First, it is

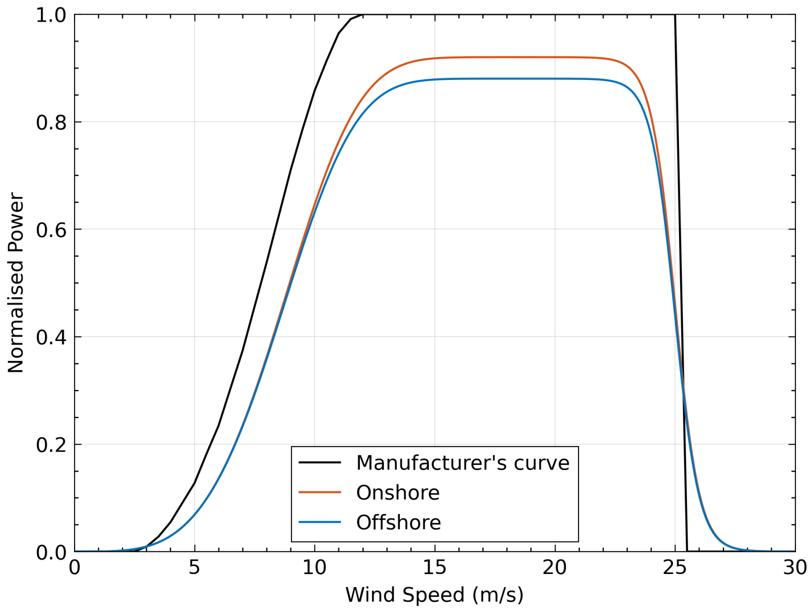

Figure 3: Effective normalised power curves for onshore and offshore farms compared against the original manufacturer's curve for a class-III wind farm.

evident that the inflection points of both curves are significantly shifted to higher wind speeds. This is due to the empirical calibration to take wake effects into consideration. While such a change is substantial, measured wind speed output from the wind farm datasets shows that this behaviour of a wind farm power curve can be expected. Second, it can be observed that the normalised rated powers of both curves are not equal to 1. This is due to the energetic availability factors applied to the curve as a constant. Note that while the normalised rated power of the offshore curve is lower than that of the onshore curve, it does not necessarily mean that the resultant power is lower for the offshore domain than onshore.

1.4. Validation

The GB-aggregated CF model comprises three major procedures, namely the bias-correction of ERA5 wind speed, the vertical extrapolation of wind speed by MLPR, and the conversion to power output by a wind farm power curve. Each of these steps represents an improvement to what is considered the *baseline* scenario where a standard approach is taken i.e., using uncorrected ERA5 wind speed data, vertical extrapolation of wind speed by a log-

arithmic profile, and conversion to power output by a manufacturer's power curve. Hence, the validations of the resultant GB-aggregated CF under these two scenarios against wind power generation data from National Grid [7] are compared in Figure 4 and the incremental changes in error metrics with respect to the implementation of each major component of the GB-aggregated CF model are presented in Table 1. It is evident that the approach taken in this study for each major process resulted in an incremental reduction in error. The most significant improvement of the model stems from the application of the wind farm power curve model, however, it also means that the resultant CF time series is highly sensitive to this process. All in all, compared to the baseline condition, the approach taken by this study to model the GB-aggregated CF time series resulted in an increase of accuracy by over 60%

	**RMSE**	**MAE**	**Bias**	**Pr**
**Baseline**	0.201	0.169	0.168	0.916
**+ Bias correction**	0.191	0.155	0.152	0.917
**+ MLPR**	0.167	0.131	0.125	0.923
**+ Farm power curve**	0.092	0.065	0.025	0.923

Table 1: Validation of the modelled GB-aggregated CF against CF based on measured power output from National Grid [7]. Each line corresponds to the addition of each step of the model as opposed to the baseline case i.e., bias correction against no bias correction, MPLR against log-law, and a wind farm power curve against a manufacturer's power curve.

2. Extreme value analysis of demand-net-wind time series

2.1. Electricity demand model

Due to limited availability of electricity demand data in the temporal domain and the assumption of a constant wind farm distribution, it was necessary to model electricity demand. The approach taken in this study is based on Bloomfield et al.'s [2] weather-dependent multiple linear regression model, which relates electricity demand to temporal variable and climate variables that are provided by ERA5. Although the original model was developed at a daily time step, the multiple linear regression model in this study was constructed on an hourly time step and is given by:

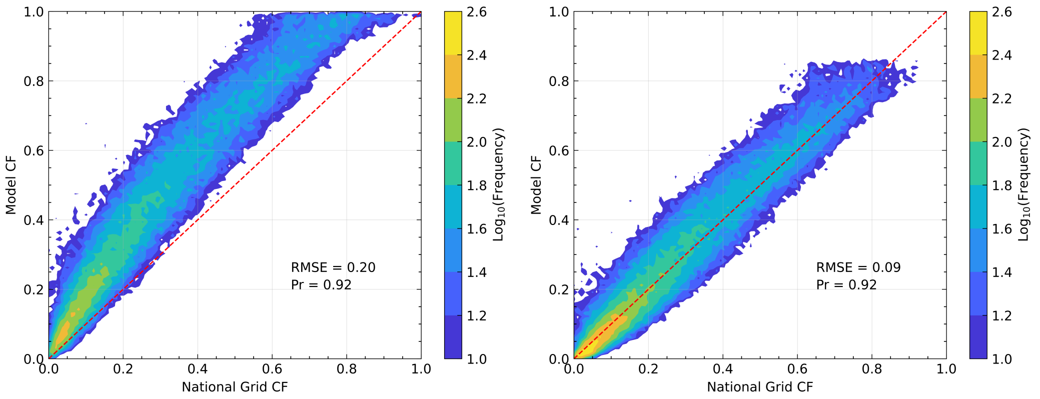

Figure 4: Validation of the modelled GB-aggregated CF against CF based on measured power output from Elexon [7] for the baseline scenario (left) and the preferred scenario (right).

$$D(t) = \alpha_0 + \alpha_1 t + \alpha_2 HDH(t) + \alpha_3 CDH(t) + \sum_{i=4}^{10} \alpha_i \cdot dayofweek(i,t) + \sum_{j=11}^{34} \alpha_j \cdot hourofday(j,t) + \sum_{k=35}^{38} \alpha_k \cdot season(k,t). \quad (3)$$

In this model, $D(t)$ refers to electricity demand at an hourly time step t. α_i's are the coefficients of regression where $\alpha_0 + \alpha_1 t$ represent the background trend of electricity demand to allow the gradual change over time due to socio-techno-economical factors. α_4 to α_{10}, α_{11} to α_{34}, and α_{35} to α_{38}, correspond to the functions $dayofweek(i,t)$, $hourofday(j,t)$, and $season(k,t)$ respectively, each of which is equal to 1 if time step t corresponds to a temporal characterisation of type i, j or k, and is equal to 0 otherwise. The remaining terms α_2 and α_3, represent the dependence of demand on weather with $HDH(t)$ (heating degree hours) and $CDH(t)$ (cooling degree hours) respectively. These two functions take the form:

$$HDH(t) = max[15.5°C - T(t), 0], \quad (4)$$

$$CDH(t) = max[T(t) - 22.0°C, 0], \quad (5)$$

where $T(t)$ is the country-average hourly temperature, calculated from all ERA5 onshore grid boxes.

The described multiple linear regression model was trained on ERA5 reanalysis temperature data against observed electricity demand data from National Grid [7] from 2010-2018 and validated from 2019-2021. The regression coefficients and the validation results are presented in Figures 6 and 5 respectively. First, it can be seen in Figure 6 that the regression coefficients expectedly represent the variation of demand with each of the three temporal functions, where demand is higher during weekdays, daytime, and winter and autumn. It should also be noted that α_1 is relatively small but non-zero, as it reflects the gradual long-term trend of electricity demand. Furthermore, as evidenced from the scatterplot, the general agreement between modelled and actual electricity demand is adequate, while the Pearson R and RMSE values of 0.93 and 2.76 GW are within the ranges of those found by Bloomfield et al. [2]. However, it should be noted that the underestimation of demand in the upper range represents a significant limitation of the model.

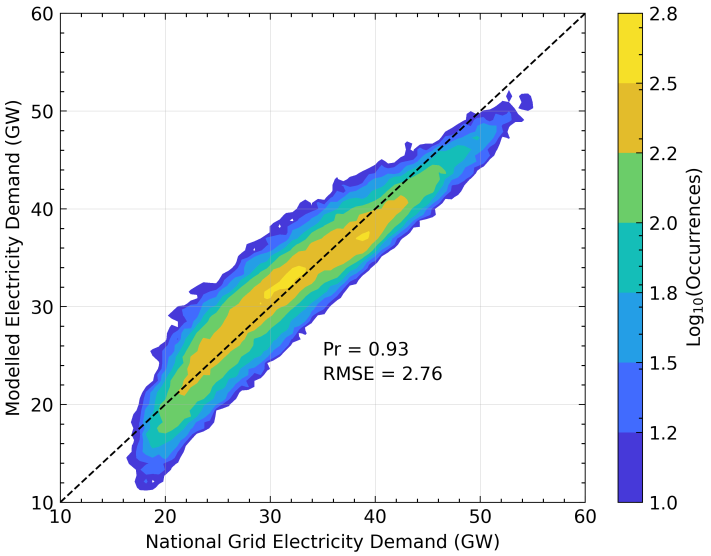

Figure 5: Modelled electricity demand plotted against measured electricity demand from National Grid [7]

References

[1] P. Potisomporn, T. A. Adcock, C. R. Vogel, Evaluating ERA5 Reanalysis Predictions of Low Wind Speed Events Around the UK, Preprint

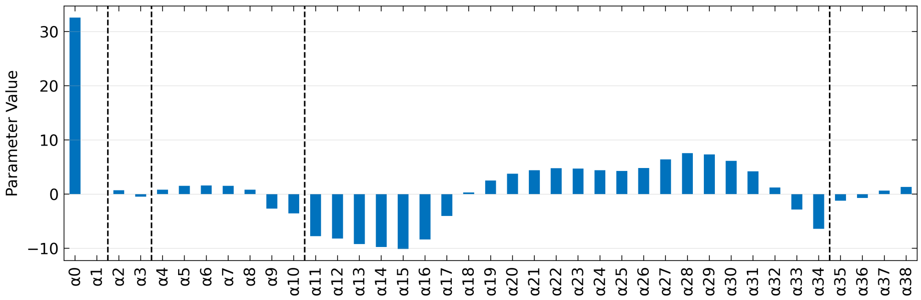

Figure 6: Regression coefficients of the weather-dependent multiple linear regression model. α_0 - α_1 correspond to the long-term trend of demand, α_2 - α_3 correspond to the weather-sensitivity of demand, α_4 - α_{10} correspond to Monday to Sunday, α_{11} - α_{34} correspond to 0000 - 2300 hours, and α_{35} - α_{38} correspond to spring (MAM), summer (JJA), autumn (SON), and winter (DJF) respectively.

submitted to Energy Reports (2023).

[2] H. C. Bloomfield, D. J. Brayshaw, A. J. Charlton-Perez, Characterizing the winter meteorological drivers of the european electricity system using targeted circulation types, Meteorological Applications 27 (2020) e1858.

[3] I. E. Commission, et al., Wind turbines-part 1: design requirements, IEC 61400-1-Ed. 3.0 (2005).

[4] M. Lydia, S. S. Kumar, A. I. Selvakumar, G. E. P. Kumar (????).

[5] C. Plumley, Kelmarsh and Penmanshiel Wind Farm Data (0.0.2), 2022.

[6] D. Cevasco, S. Koukoura, A. Kolios, Reliability, availability, maintainability data review for the identification of trends in offshore wind energy applications, Renewable and Sustainable Energy Reviews 136 (2021).

[7] Elexon, Generation by Fuel Type, 2022.
